# Combining Paclitaxel with ABT-263 Has a Synergistic Effect on Paclitaxel Resistant Prostate Cancer Cells

**DOI:** 10.1371/journal.pone.0120913

**Published:** 2015-03-26

**Authors:** Chihuei Wang, Shih-Bo Huang, Min-Chi Yang, Yi-Tsen Lin, I-Hung Chu, Ya-Ni Shen, Yueh-Ho Chiu, Shao-Hung Hung, Lin Kang, Yi-Ren Hong, Chung-Hwan Chen

**Affiliations:** 1 Department of Biotechnology, Kaohsiung Medical University, Kaohsiung, Taiwan; 2 Department of Orthopedic Surgery, Fooyin University Hospital, Pinutung, Taiwan; 3 Graduate Institute of Clinical Medicine, National Cheng Kung University, Tainan City, Taiwan; 4 Department of Obstetrics and Gynecology, National Cheng Kung University Hospital, Tainan City, Taiwan; 5 Department of Biochemistry, Kaohsiung Medical University, Kaohsiung, Taiwan; 6 Department of Orthopedics, Kaohsiung Medical University, Kaohsiung, Taiwan; 7 Department of Orthopedics, Kaohsiung Medical University Hospital, Kaohsiung, Taiwan; Innsbruck Medical University, AUSTRIA

## Abstract

We assessed the capability of paclitaxel, one of the taxanes, to induce death in two prostate cancer lines, LNCaP and PC3. Paclitaxel drove an apoptotic pathway in LNCaP, but not in PC3 cells, in response to G2/M arrest. An examination of the levels of anti-apoptotic proteins revealed that Bcl-xl was much higher in PC3 cells than in LNCaP cells and Bcl2 could be detected only in PC3 cells, not in LNCaP cells. Knocking down Bcl-xl enhanced paclitaxel-induced apoptosis in LNCaP cells, while we were unable to knock down Bcl-xl efficiently in PC3 cells. Significantly, a comparison of ABT-263, a specific inhibitor of Bcl2 and Bcl-xl, with ABT-199, a Bcl2 selective inhibitor, disclosed that only ABT-263, not ABT-199, could induce apoptosis in LNCaP and PC3 cells. The results indicate that Bcl-xl has a protective role against paclitaxel-induced apoptosis in LNCaP and PC3 cells, and its overexpression causes the paclitaxel resistance seen in PC3 cells. Interestingly, combined paclitaxel with ABT-263 to treat LNCaP and PC3 cells demonstrated synergistic apoptosis activation, indicating that ABT-263 could enhance paclitaxel-induced apoptosis in LNCaP cells and overcome Bcl-xl overexpression to trigger paclitaxel-induced apoptosis in PC3 cells. We also observed that the activation of apoptosis in LNCaP cells was more efficient than in PC3 cells in response to paclitaxel plus ABT-263 or to ABT-263 alone, suggesting that the apoptosis pathway in PC3 cells might have further differences from that in LNCaP cells even after Bcl-xl overexpression is accounted for.

## Introduction

Acquired resistance to taxane-related chemotherapy remains a major problem for malignant tumors that show an initial therapeutic benefit from taxane treatment. Cancer cells with taxane resistance might overexpress a multidrug resistance gene coded for the P-glycoprotein pump to increase the efflux of taxane, leading to minimal intracellular taxane concentrations [1]. In addition, alteration of microtubule features, mainly by increasing the dynamic activity of the microtubules after taxane treatment, might also change responsiveness to taxanes and decrease their efficacy [2,3]. Mutations of tubulin genes in the microtubule binding site of taxanes might alter taxane binding affinity, resulting in significant loss of effectiveness [4,5]. Gain-of-function to counteract apoptotic pathways might also contribute to multidrug resistance in cancers [6,7].

The specific apoptosis regulatory pattern in cancer cells might be a crucial factor determining the sensitivity of cancer cells to multiple diverse chemotherapy agents [8]. Intrinsic or mitochondrial apoptosis usually occurs in cancer cells responding to chemotherapy-induced cell cycle arrest, including drug-induced mitotic arrest [9,10]. The Bcl2 family, consisting of three groups of proteins including anti-apoptotic proteins, pro-apoptotic proteins, and BH3-only proteins, is essential for this intrinsic apoptosis [9]. Bcl2, Bcl-xl, Mcl-1 and Bfl1 are anti-apoptotic proteins. Both Bax and Bak are pro-apoptotic proteins. BH3-only proteins include Bad, Bik, Bim, Bid, Noxa, Hrk and Bmf. The anti-apoptotic proteins all contain four conserved sequence motifs, the Bcl-2 homology (BH) domains, which include BH1, BH2, BH3 and BH4 [10]. Their role is to maintain the integrity of mitochondria for supporting cell survival. The pro-apoptotic proteins share remarkable similarity with the anti-apoptotic proteins, especially in the structural features of all four BH regions, whereas they disturb mitochondrial integrity to trigger apoptosis. Lastly, the BH3-only proteins have only a BH3 domain to share with each other and with the anti-apoptotic and pro-apoptotic proteins [11]. Interestingly, this common BH3 domain of the BH3-only proteins constitutes about 26-residue amino acids and forms an amphipathic α-helix to interact with and inactivate the anti-apoptotic proteins [12]. It possibly also transiently binds Bax and Bak for their activation [13].

Recently, several compounds have been developed as BH3 mimetics to induce apoptosis through inhibition of the anti-apoptotic proteins [12,14]. So far, the most potent inhibitors are the Bad-like BH3 mimetics, ABT-737 and its orally active analog, ABT-263 [15–17]. They bind to Bcl-2, Bcl-xl and Bcl-w with very high affinity, but with much lower affinity to Mcl-1 or Bcl2A1 [14,18]. Preclinical studies have demonstrated that both ABT-737 and ABT-263 can displace the pro-apoptotic proteins from the anti-apoptotic proteins, consistent with a BH3-mimetic mechanism of killing [19]. The apoptosis induced by ABT-737 via BAX or BAK is suggested to be an on-target activity [20]. Furthermore, the sensitivity of the cell response to the BH3 peptides of the BH3-only proteins is highly correlated with the sensitivity of the cells threatened with ABT-737 apoptosis [21]. Significantly, clinical trials of ABT-263 have been performed and some benefit has been observed, most notably in chronic lymphocytic leukemia [22]. In addition, both ABT-737 and ABT-263 can be used as tools for mechanistic studies of apoptosis [14,23]. Recently, a new ABT, ABT-199, has been developed with demonstrated selectivity specifically for Bcl2 [24].

In the current study, we explored the apoptotic pathways in prostate cancer cells in response to paclitaxel, ABT-199, ABT-263 and paclitaxel plus ABT-263, using LNCaP and PC3 cells. LNCaP and PC3 represent the androgen-response and androgen-independent prostate cancer cell line, respectively. We first found that paclitaxel caused G2/M arrest in both LNCaP and PC3 cells, but apoptosis only resulted in LNCaP cells. Moreover, we observed Bcl2 and Bcl-xl overexpression in PC3 cells compared to LNCaP cells, which have low and undetectable levels of Bcl-xl and Bcl2 respectively. The siRNA knockdown of the anti-apoptotic protein Bcl-xl increased apoptosis in LNCaP, but it could not be knockdown in PC3 cells efficiently. We compared the ability of ABT-199 and ABT-263 to induce apoptosis and found that only ABT-263, not ABT-199, could induce apoptosis. We also demonstrated that paclitaxel in combination with ABT-263 had synergistic effects on apoptosis in both LNCaP and PC3 cells. This suggests that ABT-263 might improve therapeutic outcomes for both paclitaxel-sensitive and paclitaxel-resistant prostate cancers. Activation of apoptosis was more efficient in LNCaP cells than PC3 cells in response to paclitaxel plus ABT-263 or ABT-263 alone, suggesting that the apoptosis pathway in PC3 cells might differ further from that in LNCaP cells even after Bcl-xl is accounted for.

## Materials and Methods

### Compounds

Paciltaxel (Sigma-Aldrich), ABT-199 (Selleck) and ABT-263 (Selleck) were purchased from commercial sources as indicated. The compounds were dissolved in DMSO first and diluted by culture medium in further experiments.

### Cell culture and synchronization

PC3 and LNCaP cells were purchased from the Bioresource Collection and Research Center (BCRC) in Taiwan. The cells were seeded at 4 × 10^5^–6 × 10^5^ cells per petri dish (10 cm) in RPMI 1640 with 10% fetal bovine serum and grown at 37°C under 5% CO_2_. The cells were cultured either non-synchronously or in synchronization. For synchronization, PC3 cells were seeded in serum-rich RPMI 1640 medium with 2 mM thymidine and incubated for 19 hr. The cells were released by washing three times with PBS and re-fed with fresh serum-rich medium for 8 hr. Then cells were re-fed with fresh medium containing 2 mM thymidine for 16 hr. The cells were washed by PBS three times before subsequent steps. LNCaP cells were starved in serum-free RPMI 1640 medium for 48 hr after being seeded in serum-rich medium for 24 hr.

### Flow cytometric cell cycle analysis

PC3 and LNCaP cells were treated by paclitaxel at the indicated time points following synchronization in G1/S phase. The cells were harvested by trypsinization and/or separated into adherent and detached fractions. The cells were centrifuged, washed with PBS and collected by centrifugation. The cells were fixed with ice-cold 70% ethanol for 30 min, washed with PBS and centrifuged to remove supernatant. The cells were re-suspended in PBS containing 0.05% Triton X-100 and RNase A (40 μg/mL) and incubated at 37°C for 1 hr, and propidium iodide (PI) was added to the cell suspension to a final concentration of 50 μg/mL for another 1 hr incubation. The cells were harvested by centrifugation, washed with PBS and centrifuged to remove supernatant. Finally, the cells were re-suspended by PBS and analyzed by flow cytometer (BD Biosciences) with software (BD Biosciences).

### Immunofluorescence analysis with a confocal microscope

About 5× 10^4^ PC3 and 1× 10^5^ LNCaP cells were plated on sterile 18-mm glass coverslips. Cells were synchronized under the conditions described above in the cell synchronization section. Cells were cultured with different compounds at the indicated time courses, fixed in methanol (-20°C, 10 min) and perforated with 0.1% triton X100 (25°C, 2 min). Cells were blocked in 3% bovine serum albumin-TBS and then stained with primary α-Tubulin monoclonal antibody (Sigma-Aldrich) followed by secondary antibodies, conjugated with fluorophore (Invitrogen). The coverslips were mounted with antifade reagent with DAPI (Invitrogen) upside down on glass slides. Confocal microscopy images were obtained using a confocal microscope system (Olympus) and amplified one hundred times.

### Apoptosis Assay

About 1×10^5^ PC3 cells or 2×10^5^ LNCaP cells were seeded on a 10 cm petri dish. Both PC3 and LNCaP cells were synchronized and exposed to the indicated compound treatments for 48 hr. The cells were harvested by trypsinization and separated into adherent and detached fractions. The cells were resuspended and stained in 100 μl of Annexin V binding buffer with 100 μg/mL propidium iodide and 100 μg/mL FITC-conjugated Annexin V antibody (Strong Biotech) for 15 min at room temperature before FACS analysis.

### Immunoblotting

Cells were lysed in lysis buffer (250 mM NaCl, 1 mM DTT, 0.1% NP-40, 1 mM EDTA, 10 mM β-glycerophosphate, 0.1 mM Na_3_VO_4_H, 1 tablet protease inhibitor cocktail, 1 mM NaF, 50 mM HEPES, pH7.4, for 50 ml). Protein concentration was determined by a BCA Protein Assay Kit (Pierce). About 100 μg of protein per well was subjected to SDS-PAGE. After electrophoresis, the proteins were transferred to a nitrocellulose membrane. The transferred membranes were blocked in 5% (w/v) nonfat dry milk or 5% (w/v) BSA in TBS (0.5 M NaCl, 20 mM Tris-HCl, pH 7.4) with 0.1% (v/v) Tween 20 and probed for the first antibody, followed by incubation with a secondary antibody conjugated with horseradish peroxidase (anti-rabbit, anti-mouse; Jackson ImmunoResearch) and visualization with a detection kit (Pierce) by photographic film development. The first antibodies used in this study were as follows: anti-actin antibody (Millipore), anti-glyceraldehyde-3-phosphate dehydrogenase (GAPDH) and -α-tubulin antibody (GeneTex), anti-Bcl-xl antibody (Abcam), Anti-Bak,-Bax,-Bcl2,-Bim,-caspase 3,-caspase 3(A),-caspase 7(A),-Mcl-1,-poly(ADP-ribose) polymerase (PARP) and-PARP(C) antibody (Cell Signaling). Immunoblot images were quantitated by quantitative software (NIH).

### Coimmunoprecipitation

Total cell lysates of paclitaxel-treated or control LNCaP or PC3 cells were prepared in Chaps buffer (5mM MgCl_2_, 137mM NaCl, 1mM EDTA, 1mM EGTA,1% Chaps, 20mM Tris–HCl (pH 7.5)), and protease inhibitors. About 500 μg of proteins were immunoprecipitated with Bcl-xl antibody (Abcam) at 4°C for 2 h. Immunoprecipitates were captured by 50 μl of the slurry of protein A-magnetic beads (Millipore) in Chaps buffer at 4°C for overnight. Immunoprecipitates were then recovered by magnetic stand and washed three times in 1% Chaps buffer. Immunoprecipitates were eluted by SDS-PAGE sample buffer, were subjected to SDS-PAGE and then immunoblotting.

### Bcl-xl, Bim and Mcl-1 knock down by siRNA

PC3 or LNCaP cells were seeded at 4 × 10^5^–6 × 10^5^ cells per petri dish (10 cm) for 24 hours in 7 ml RPMI 1640 with 10% fetal bovine serum and grown at 37°C under 5% CO_2_. Bim (Qiagen), Bcl-xl and MCl-1 (Invitrogen) siRNA was preincubated in 1 ml culture medium without serum before transfection, and then 40 μl transfection reagent (Qiagen) was added to the same culture medium, mixed by vortex and incubated for 5~10 min at room temperature to allow the formation of transfection complexes. The final siRNA concentration was about 5 to 10 nM after adding the complexes drop-wise to PC3 and LNCaP cells. After 24 hours, PC3 and LNCaP cells were treated with 50 nM of paclitaxel and harvested at the indicated time courses.

### Statistical analysis

A paired t-test was used to show the statistical significance of the results using SigmaPlot 10. **P < 0.01 was considered significant. Data from two independent experiments were analyzed.

## Results

### Paclitaxel led to mitotic arrest at G2/M in both PC3 and LNCaP cells

**Fig 1:**
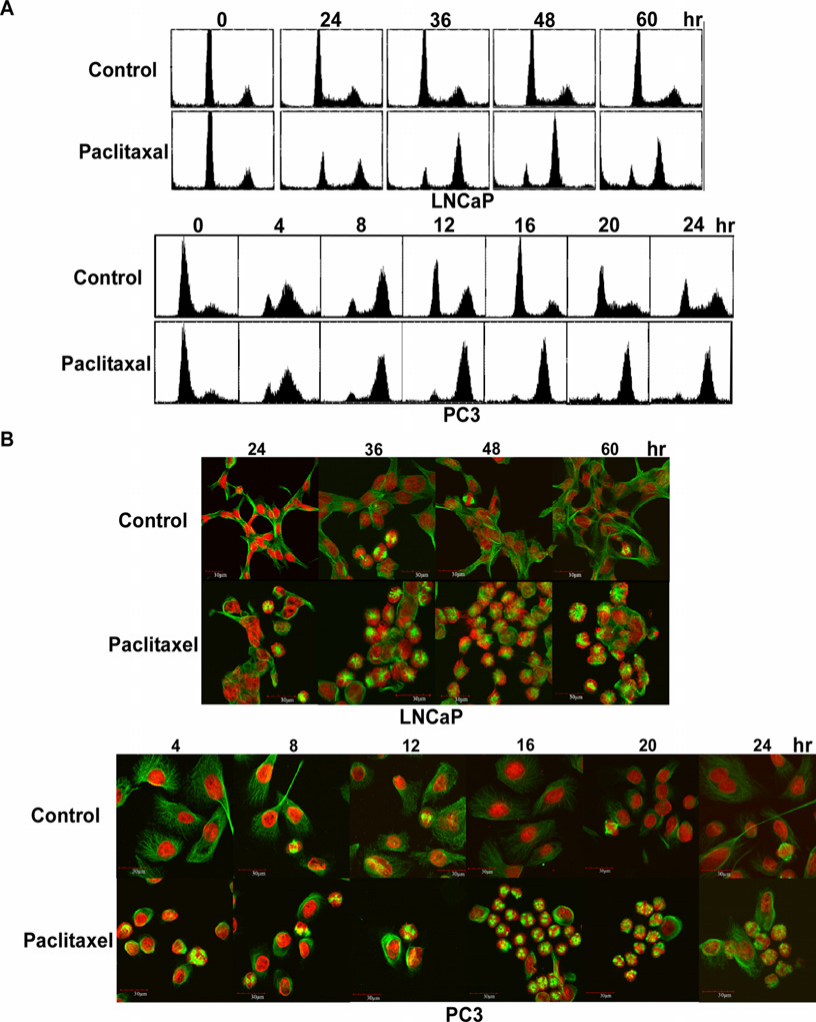
Paclitaxel induced cell cycle arrest at G2/M in LNCaP and PC3 cells. (A) Flow cytometric analyses of synchronized PC3 or LNCaP cells treated by paclitaxel through the indicated time courses. Analyses were in triplicate and representative histograms are shown. X- and Y-axes represent DNA content and cell numbers, respectively. (B) Immunofluorescence micrograph of microtubules from synchronized PC3 or LNCaP cells treated by paclitaxel over the indicated time courses. Analyses were duplicated and representative immunofluorescence micrographs are shown. α-Tubulin was shown by green color. DNA was shown by red color.

Synchronized PC3 or LNCaP cells at G1/S were treated with 50 nM of paclitaxel over different time courses and harvested for cell cycle analysis with flow cytometry. We found that the cell cycle proceeded in PC3 cells normally through S phase and was arrested at the time point between 8 and 12 hr (Fig. 1A). Because of its slow proliferation rate, LNCaP cells took longer than PC3 to respond to compound treatment, showing cell cycle arrest between 36 and 48 hr (Fig. 1A).

Then we monitored the behavior of both microtubules and chromosomes by immunofluorescence imaging with a confocal microscope over the same time course. We observed that paclitaxel affected microtubules at 4 hr and 8 hr in PC3 cells, showing abnormally dense microtubule distribution around the nucleus (Fig. 1B). These abnormal microtubules lost the capacity to perform normal spindle assembly, resulting in dense multipolar spindles at 12 hr (Fig. 1B). The effect of paclitaxel on LNCaP cells resembled the effect on PC3 cells (Fig. 1B). Nevertheless, due to the long duplication time of LNCaP, the abnormal spindle formation in LNCaP cells occurred much later than in PC3 cells (Fig. 1B).

Our results suggested that cells treated by paclitaxel could still go forward through G2 and probably enter G2/M to form the defective spindle-chromosome complex that then caused cell cycle arrest at this phase.

### Paclitaxel drove an apoptotic response through the caspase-dependent apoptotic pathways in LNCaP, but not in PC3 cells,in response to G2/M arrest

**Fig 2:**
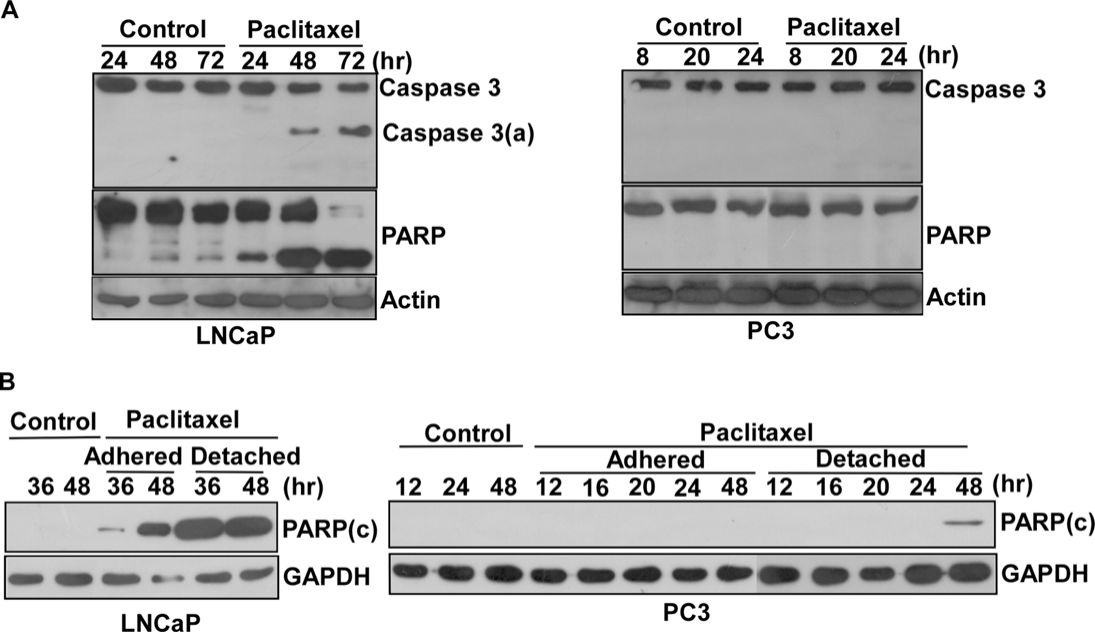
Caspase-dependent apoptotic pathways were seen in LNCaP, but not in PC3 cells, in response to G2/M arrest. (A) Immunoblot analysis of cell lysates from synchronized LNCaP and PC3 cells treated by paclitaxel over the indicated time courses for the detection of caspase 3 and PARP with its degradation product. (B) Immunoblot analysis of cell lysates from the adhered or detached fractions of LNCaP or PC3 cells after paclitaxel treatment over time courses for the detection of the cleavage form of PARP. Experiments were repeated three times and representative results are shown. PARP (c): the cleavage form of PARP.

Paclitaxel caused cell cycle arrest at G2/M, around 8–12 hr in PC3 cells and 36–48 hr in LNCaP cells. Next, we investigated the apoptotic responses triggered by paclitaxel by assessing the activation of caspase 3 and the degradation level of PARP. Our results revealed that paclitaxel caused the activation of caspase 3 and PARP degradation in LNCaP cells to appear first at 48 hr, right after G2/M arrest, and reached a significant level at 72 hr (Fig. 2A). In contrast, we detected neither the activation of caspase 3 nor the degradation of PARP in PC3 cells by paclitaxel at G2/M arrest (Fig. 2A). Thus, we concluded that paclitaxel activated an apoptotic response highly corresponding to its effect on G2/M arrest in LNCaP cells, but not in PC3 cells.

### Paclitaxel caused the death of PC3 cells only in detached status

We found that paclitaxel did not activate an apoptotic response corresponding to the G2/M arrest in PC3 cells. What is the fate of the PC3 cells stuck in this phase? After paclitaxel treatment, we observed that some LNCaP or PC3 cells detached from the bottom of the culture dish. Thus we harvested cells after incubation with the compounds over different time courses, and collected and analyzed the adhered and detached cells separately. We looked into the degradation of PARP in the adhered and detached fractions of LNCaP and PC3 cells at different time courses. The cleavage form of PARP appeared in the adhered and detached fractions of LNCaP cells (Fig. 2B). This form only appeared in the detached fraction of PC3 cells at 48 hr (Fig. 2B).

We also performed Annexin-V-FITC/PI staining to evaluate cell death in LNCaP or PC3 cells. Most adhered PC3 cells remained alive, while the adhered fraction of LNCaP cells showed a significant percentage falling into early apoptosis (S1 Fig.). For the detached fraction, LNCaP and PC3 cells appeared in late apoptosis and necrosis (S1 Fig.).

We concluded that the timing of the early apoptosis in the adhered fraction of LNCaP cells corresponds to G2/M arrest while the PC3 cells did indeed display a resistance phenotype to paclitaxel treatment. The death in the detached fractions of LNCaP and PC3 seemed related to cell detachment as well as cellular stress caused by paclitaxel.

### Expression of Bim, Bak, Bax, Bcl2, Bcl-xl and Mcl-1 in LNCaP and PC3 cells after treatment with paclitaxel

**Fig 3:**
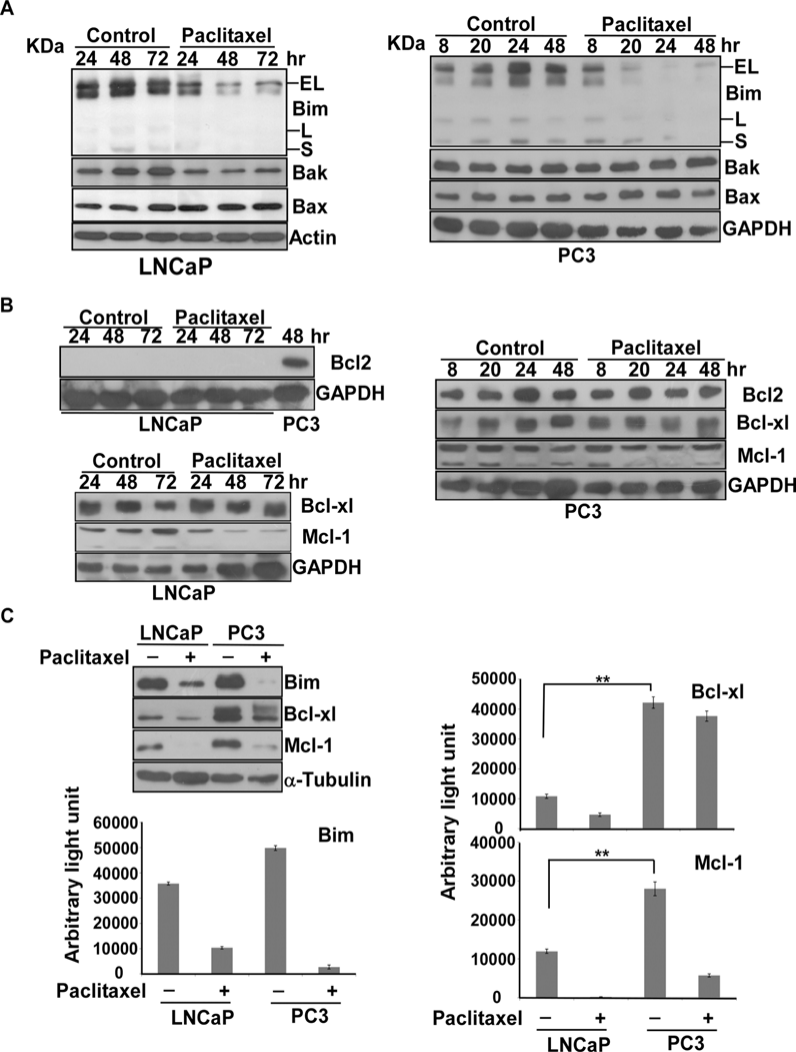
The expression of BH3-only protein, pro-apoptotic proteins and anti-apoptotic proteins in LNCaP or PC3 cells after treatment with paclitaxel. (A) The expression of BH3-only protein Bim and pro-apoptotic proteins including both Bak and Bax, in LNCaP and PC3 cells. (B) The expression of anti-apoptotic proteins including Bcl2, Bcl-xl and Mcl-1 in LNCaP or PC3 cells. Immunoblot analysis of cell lysates from LNCaP or PC3 cells treated by paclitaxel over time as indicated. Experiments were repeated three times and representative results are shown. (C) Comparison of Bim, Bcl-xl and Mcl-1 between LNCaP and PC3 cells with/without paclitaxel treatment. Immunoblot analysis of cell lysates from LNCaP or PC3 cells treated for 48 hr. Experiments were repeated three times and representative results are shown. Immunoblot images of Bim, Bcl-xl, Mcl-1 and α-tubulin in LNCaP and PC3 with/without paclitaxel treatment were quantitated by ImageJ. The readouts, defined as arbitrary light units, of Bim, Bcl-xl and Mcl-1 normalized to the readout of α-tubulin are shown on the right side. (**) statistical significance between two readouts.

To ask why caspase 3-dependent apoptosis occurs in LNCaP, but not in PC3 cells, in response to G2/M arrest, we checked the protein level of Bim, Bak, Bax, Bcl2, Bcl-xl and Mcl-1. Our results showed that the protein level of Bim decreased over time after paclitaxel treatment in LNCaP and PC3 cells (Fig. 3A). In contrast, the protein level of Bak, Bax, Bcl-xl and Mcl-1 did not change significantly under the same conditions (Figs. 3A and B). We could not detect any Bcl2 proteins in the LNCaP cells (Fig. 3B). Interestingly, PC3 cells expressed a significant amount of Bcl2 proteins with or without paclitaxel treatment (Fig. 3B). We then compared the protein level of Bim, Bcl-xl and Mcl-1 between LNCaP and PC3 cells. The results disclosed that the expression of Bcl-xl and Mcl-1 in PC3 cells is much higher than in LNCaP cells (Fig. 3C). Therefore, the overexpression of Bcl2, Bcl-xl and Mcl-1 in PC3 cells might contribute to their paclitaxel resistance.

The Bim level of LNCaP or PC3 cells decreased dramatically after paclitaxel treatment, and correlated with cell death (Fig. 3A). We asked why the level of Bim was significantly reduced after paclitaxel treatment. Again, we collected and analyzed data for the adhered and detached cells separately. Reduced Bim only appeared in the detached fraction, while it remained constant in the adhered fraction in both LNCaP and PC3 cells after paclitaxel treatment (S2 Fig.). Thus, the reduced Bim level might not be related to its role in the apoptotic pathway.

Next, we asked if Bim involves in paclitaxel-induced apoptosis pathway in prostate cancers by RNAi knockdown and coimmunoprecipitation, since Bim is a main BH3-only protein required for paclitaxel-induced apoptosis in breast cancers [25]. Different from breast cancers, our results demonstrated that Bim knockdown cannot affect paclitaxel-induced apoptosis in LNCaP cells (S3 Fig.). Moreover, no significant interaction between Bim and Bcl-xl was observed in IP (S3 Fig.). These results suggested that Bim might not be an essential BH3-only protein responsible for paclitaxel-induced apoptosis in prostate cancers.

### Bcl-xl or Mcl-1 knockdown enhances apoptosis in LNCaP cells, but not PC3 cells

**Fig 4:**
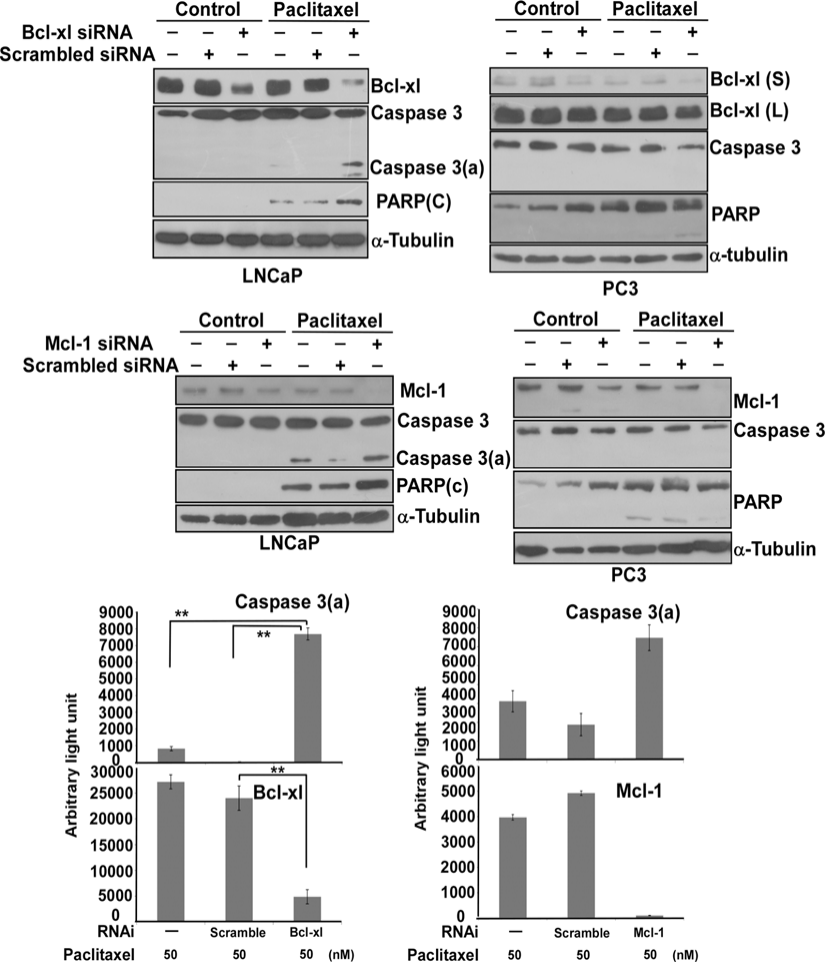
The knockdown effect of Bcl-xl or Mcl-1 in LNCaP or PC3 cells. Bcl-xl and Mcl-1 knockdown caused increased apoptosis as assessed by the activation of caspase 3 and PARP degradation in LNCaP cells, but not in PC3 cells. LNCaP or PC3 cells were transiently transfected with Bim, Bcl-xl or Mcl-1 siRNA or scrambled siRNA for 24 hr. Cells were treated with/without 50 nM of paclitaxel for 48 hr, and then subjected to immunoblot analysis. Caspase 3(a): the active form of caspase 3. PARP(c): the cleavage form of PARP. Bcl-xl(s): the short exposure image of Bcl-xl. Bcl-xl (l): the long exposure image of Bcl-xl. Experiments were repeated three times and representative results are shown. The respective readouts of the immunoblot images of Bcl-xl, Mcl-1 and caspase 3(a) normalized to α-tubulin in LNCaP cells are shown at the bottom. Only knockdown with/without paclitaxel treatment was quantitated. (**)statistical significance between two readouts.

To ask if the overexpression of Bcl2, Bcl-xl and Mcl-1 in PC3 cells might contribute to their paclitaxel resistance, we first explored which of them might engage in the apoptotic pathway. The results demonstrated that Bcl-xl knockdown by siRNA significantly increased the activation of caspase 3 and the degradation of PARP in LNCaP cells (Fig. 4). Mcl-1 knockdown also increased the activation of caspase 3 and the degradation of PARP in LNCaP cells, but its effect was not as good as Bcl-xl knockdown (Fig. 4). These results suggested that Bcl-xl might be a major factor affecting the paclitaxel-induced apoptotic pathway in LNCaP cells.

We then performed the same assays in PC3 cells. We had difficulty knocking down Bcl-xl efficiently in PC3 cells, probably due to the high level of this protein (Fig. 4). In contrast, the protein level of Mcl-1 was significantly reduced by its siRNA but this knockdown had no effect on the activation of caspase 3 and the degradation of PARP in PC3 cells (Fig. 4).

### The overexpression of Bcl-xl contributed most to the paclitaxel resistant phenotype in PC3 cells

**Fig 5:**
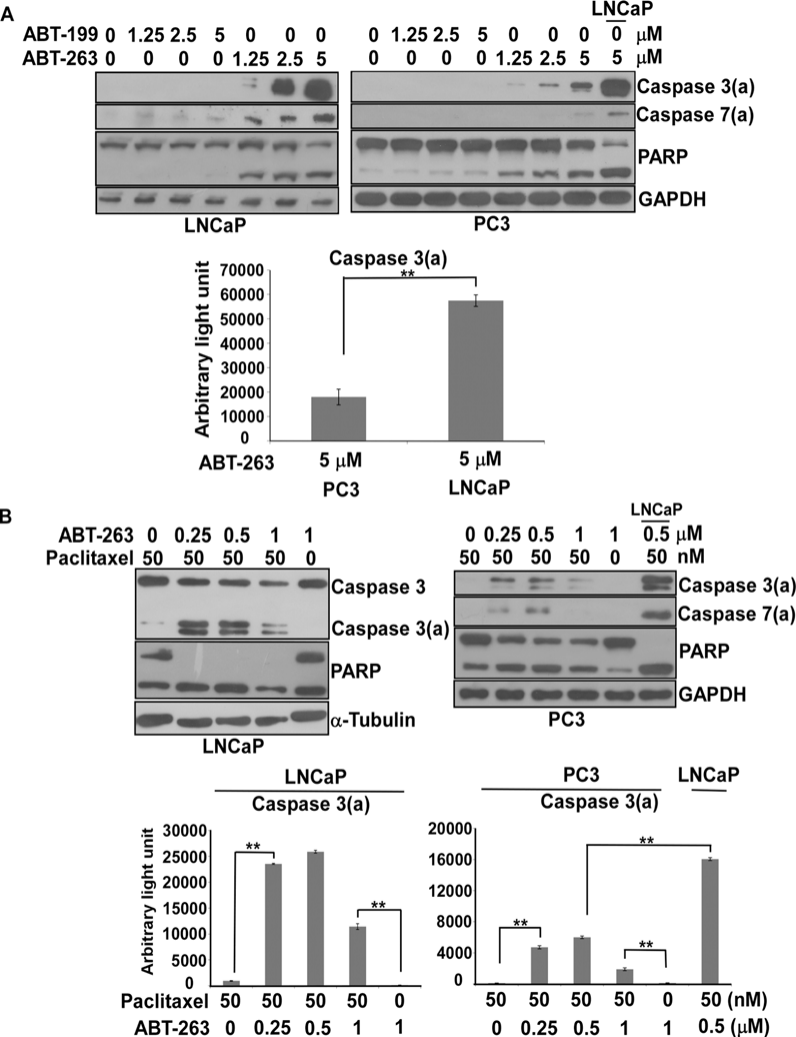
The effect of ABT-199, ABT-263 or ABT-263 in combination with paclitaxel on LNCaP or PC3 cells. (A) ABT-263, but not ABT-199, could effectively trigger PARP degradation in LNCaP and PC3 cells in a dose-dependent manner, but it could only activate caspase 3 to a detectable level in LNCaP cells. Immunoblot analysis of cell lysates from LNCaP or PC3 cells treated by ABT-199 or ABT-263 alone for 48 hr at various concentrations were assayed as indicated. Caspase 3(a): the activated form of caspase 3. Caspase 7(a): the activation form of caspase 7. Experiments were repeated three times and representative results are shown. The readouts of caspase 3(a) normalized to GAPDH between LNCaP and PC3 cells treated with 5μM of ABT-263 are shown at the bottom. (**)statistical significance between two readouts. (B) The combination of 50 nM of paclitaxel with various concentrations of ABT-263 had a synergistic effect on apoptosis in both LNCaP and PC3 cells. The efficacy of ABT-263 in combination with paclitaxel in LNCaP cells was higher than in PC3 cells. Immunoblot analysis of cell lysates from LNCaP or PC3 cells treated by paclitaxel in combination with ABT-263 or ABT-263 alone for 48 hr at various concentrations were assayed as indicated. Caspase 3(a): the activated form of caspase 3. Caspase 7(a): the activation form of caspase 7. Experiments were repeated three times and representative results are shown. The respective readouts of caspase 3(a) normalized to internal control, α-tubulin or GAPDH in LNCaP or PC3 with LNCaP are shown at the bottom. (**) statistical significance between two readouts.

Since we could not efficiently knock down Bcl-xl to evaluate its effect on paclitaxel-induced apoptosis in PC3 cells, our alternative approach was to use ABT-263 for chemical knockdown of Bcl2 and Bcl-xl simultaneously. Moreover, we used ABT-199, a specific inhibitor of Bcl2, to distinguish between Bcl2 and Bcl-xl in PC3 cells. Using ABT-263 induced the degradation of PARP and the activation of caspase 3 in both LNCaP and PC3 cells (Fig. 5A). However, the effect of ABT-263 on LNCaP cells was about 3-fold greater than for PC3 in terms of caspase 3 activation (Fig. 5A). Significantly, ABT-199 did not display any apoptotic effect on either LNCaP and PC3 cells, ruling out an anti-apoptotic role of Bcl2 in PC3.

Collectively the results suggest that Bcl-xl is the most important factor in apoptosis and its overexpression contributes to the resistance phenotype to paclitaxel treatment in PC3 cells. Moreover, we also found that PC3 cells display partial resistance to ABT-263 treatment if the response of LNCaP to the same treatment is considered as a full response.

### The combination of ABT-263 with paclitaxel demonstrated a synergistic effect on apoptosis in both LNCaP and PC3 cells

We have shown that ABT-263 alone can drive paclitaxel-sensitive and paclitaxel-resistant cells toward apoptosis, although the paclitaxel-sensitive LNCaP cell line shows better effects than the paclitaxel-resistant PC3 cell line. We further evaluated the combination effect of ABT-263 with paclitaxel. Significantly, our results demonstrated that ABT-263 in combination with paclitaxel had a synergistic effect on apoptosis in both LNCaP and PC3 cells (Fig. 5B). However, the efficacy of this combination treatment was greater in LNCaP cells than in PC3 cells (Fig. 5B).

The synergy between paclitaxel and ABT-263 for the activation of apoptosis clearly demonstrated that ABT-263 can counteract Bcl-xl in paclitaxel-sensitive and paclitaxel-resistant prostate cancer cells. Targeting anti-apoptotic proteins has the potential to improve chemotherapy for prostate cancers. However, the difference in apoptosis activation between LNCaP and PC3 cells for ABT-263 alone or ABT-263 in combination with paclitaxel suggested that the apoptosis pathway in PC3 cells might differ further from that in LNCaP cells even after Bcl-xl is accounted for.

## Discussion

In our study paclitaxel triggered early apoptosis in the adherent fraction of LNCaP cells in response to G2/M arrest. In contrast, early apoptosis could not be detected in the same fraction of PC3 cells over the whole time course, suggesting that PC3 cells do not respond to G2/M arrest to initiate this death program. Interestingly, PC3 cell death occurred in a detached status after paclitaxel treatment mainly through necrosis. Moreover, the Bim level only decreased in the detached fraction of both cells after paclitaxel treatment. Whether the decrease of Bim levels is related to cell death in detached conditions still remains to be resolved. Another study indicated that Bim knockdown in prostate and breast cancer cells caused cell detachment and ultimately apoptosis [26]. However, we did not observe the phenomenon described in that report.

Whether cell detachment might happen *in vivo* in anti-mitotic therapies is an interesting issue that remains to be determined. Logically, cancer cells surrounded by connective tissues might be very hard to detach from cancer tissue after anti-mitotic chemotherapy. Thus, cell death in response to G2/M arrest in an adhered status becomes the critical point for judging whether cells are sensitive or resistant to treatment with paclitaxel. In the current study, we demonstrated that LNCaP cells exhibit sensitivity to paclitaxel treatment, whereas PC3 cells show resistance.

We also demonstrated that overexpression of Bcl-xl might contribute to the apoptosis-resistant phenotype of PC3 cells. It seems that the apoptosis signal induced by paclitaxel treatment might be not sufficient to counteract the overexpression of anti-apoptotic proteins such as Bcl-xl in PC3 cells. This speculation is further supported by the combination of paclitaxel with ABT-263 which had synergistic effects on the activation of caspase 3 and the degradation of PARP compared to paclitaxel or ABT-263 alone.

Indeed, one study has pointed out that overexpression of Bcl-xl in high-grade prostate carcinoma is associated with a hormone-refractory phenotype [27]. PC3 is one of the best representative hormone-refractory cell lines, and paclitaxel-related compounds such as docetaxel and cabazitaxel have shown some benefits in clinical trials for hormone-refractory or castration-resistant prostate cancer (CRPC). Whether overexpression of Bcl-xl is involved in taxane resistance in CRPC is an interesting issue which needs to be addressed.

Moreover, our results demonstrated a differential response in apoptosis activation between LNCaP and PC3 cells using paclitaxel in combination with ABT-263 or ABT-263 alone (Fig. 5). We tried increasing the concentration of ABT-263 to 10 μM in single agent format, resulting in off-target effects (data not shown). We considered that a concentration of ABT-263 up to 5 μM should be enough to repress all Bcl2 and Bcl-xl in either LNCaP or PC3 cells. In this respect LNCaP and PC3 represented sensitive and partially resistant cell types, respectively, for inhibitors of anti-apoptotic proteins like ABT-263.

Since ABT-263 and ABT-737 have no capacity to interact with Mcl-1, factors affecting Mcl-1 levels might be the major determinants governing the sensitivity of both compounds [28]. For example, the stress protein BAG3 can stabilize Mcl-1, resulting in increasing ABT-737 resistance in several cancer types including prostate cancers [29]. Boiani et al. reported that the expression of Mcl-1 was not significantly different between LNCaP and PC3, which matches our observations (Fig. 3). However, they reported that both LNCaP and PC3 were ABT-resistant cells, which is inconsistent both with our findings and the findings of Parrondo et al. [30]. If the level of Mcl-1 is not the determining factor in the differential response of LNCaP and PC3 cells to ABT-263 treatment, one recent study may have demonstrated a different mechanism where ABT-737 induced protective autophagy to escape apoptosis in hepatocellular carcinoma with overexpression of Bcl2 [31]. This alternate mechanism seems not to apply in our case because ABT-263 did induce apoptosis, in combination with paclitaxel or as a single agent, in PC3 cells.

Although we do not have the full explanation for the differential sensitivity to ABT-263 in LNCaP and PC3 cells, the explanation might lie with the mitochondria. The inter-membrane spaces of mitochondria contain several apoptogenic factors including Cyto c, apoptosis inducing factor (AIF), Smac/DIABLO and endonuclease G [32]. Usually the apoptotic signals caused by various cellular stresses alter mitochondrial permeability, leading to the release of these apoptogenic proteins to activate the complex processes that result in cell death. The most common model proposes that inhibition of the anti-apoptotic proteins releases Bak and Bax to form a channel, allowing Cyto c or other apoptogenic factors to leak out. However, the clear mechanism by which Bak and Bax are activated to form a pore for mitochondrial outer-membrane permeabilization (MOMP) still remains to be elucidated.

Several models have been proposed to describe how Bak and Bax promote MOMP, including proteinaceous channels, lipidic pores and mitochondrial dynamics [33,34]. Cells sensitive or resistant to ABT-263 or paclitaxel may show differences in MOMP. It is highly possible that unidentified proteins on the mitochondrial outer membrane might cooperate with Bak and Bax to determine cellular sensitivity to ABT-263 and paclitaxel. Further research using ABT-737 or ABT-263 to treat LNCaP and PC3 cells in combination with a mitochondrial proteomics approach might help resolve the issue.

In sum, our study demonstrated that the LNCaP and PC3 cancer cells constitute a paclitaxel sensitive and resistant prostate cancer cell model, respectively. Part of the reason for the contrasting LNCaP and PC3 response to paclitaxel is the differential expression of Bcl-xl anti-apoptotic proteins. In addition, the differential activation of apoptosis in response to ABT-263 alone or ABT-263 plus paclitaxel suggests further differences in apoptosis pathways between LNCaP and PC3 cells.

## Supporting Information

S1 Fig Paclitaxel caused different cell death patterns in LNCaP and PC3 cells. Annexin-V-FITC staining evaluated the proportion of cells in normal, necrosis, early apoptosis and late apoptosis in the adhered or detached fractions of LNCaP or PC3 cells after paclitaxel treatment for 48 hr.
(TIF)

S2 Fig Bim instability was accompanied with cell detachment. Immunoblot analysis of cell lysates from the adhered or detached fraction of LNCaP cells or PC3 cells after paclitaxel treatment through the time courses as indicated, for the detection of Bim.
(TIF)

S3 Fig Bim was not an essential factor responsible for paclitaxel-induced apoptosis in LNCaP cells. A) Immunoblot analysis of cell lysates from Bim knockdown of LNCaP cells after paclitaxel treatment. B) Immunoblot analysis of immunoprecipitates of Bcl-xl antibody for the cell lysates of LNCaP cells or PC3 cells treated by paclitaxel. IP: immunoprecipitation. IB: immunoblotting.
(TIF)
