# Supplementary figures and images for: Combining Paclitaxel with ABT-263 Has a Synergistic Effect on Paclitaxel Resistant Prostate Cancer Cells
Source: PLoS One. 2015 Mar 26;10(3):e0120913. doi: 10.1371/journal.pone.0120913 (PMC4374961; doi:10.1371/journal.pone.0120913)

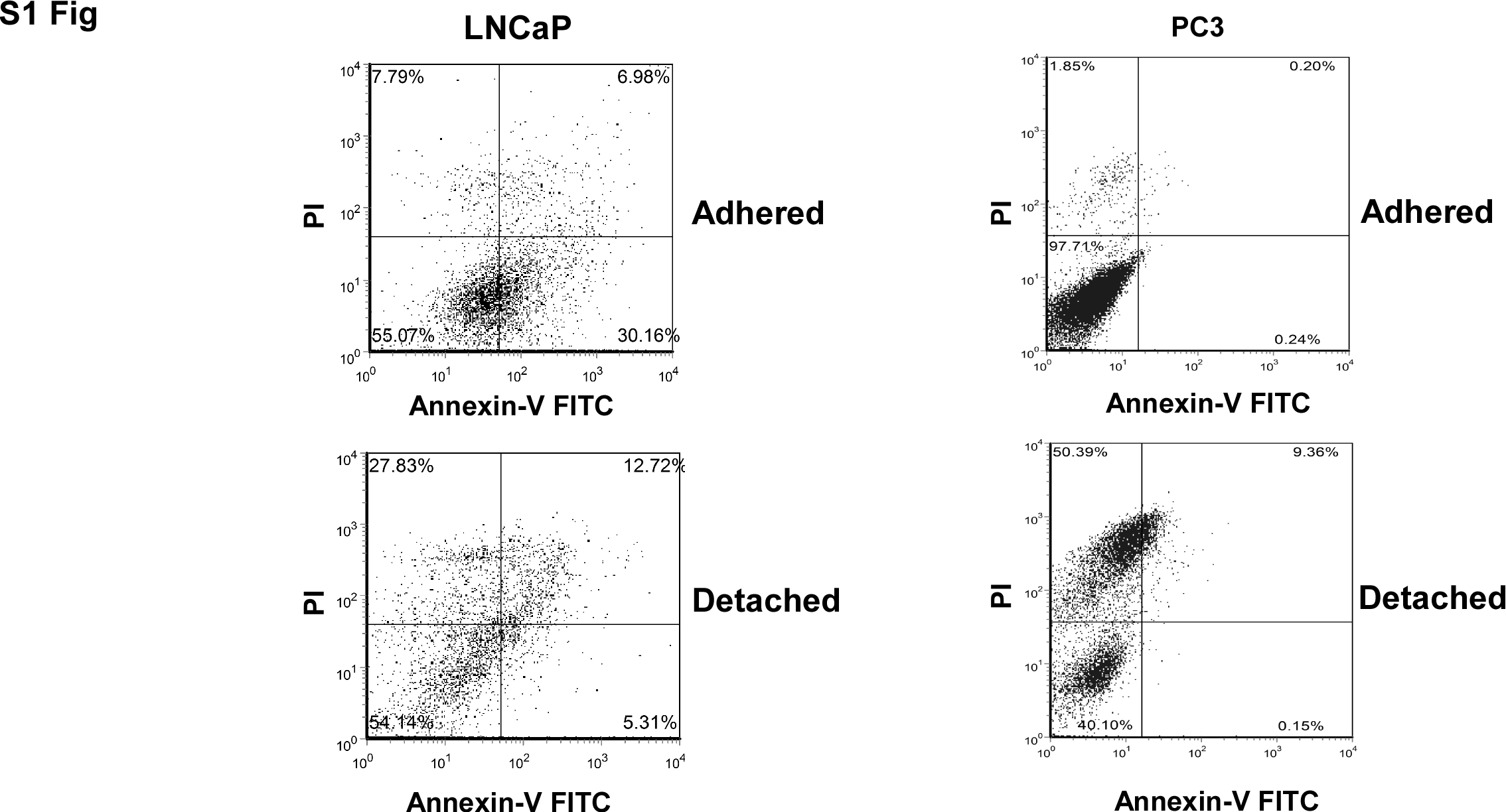

Supplement: S1 Fig — Annexin-V-FITC staining evaluated the proportion of cells in normal, necrosis, early apoptosis and late apoptosis in the adhered or detached fractions of LNCaP or PC3 cells after paclitaxel treatment for 48 hr. (TIF) [file pone.0120913.s001.tif]

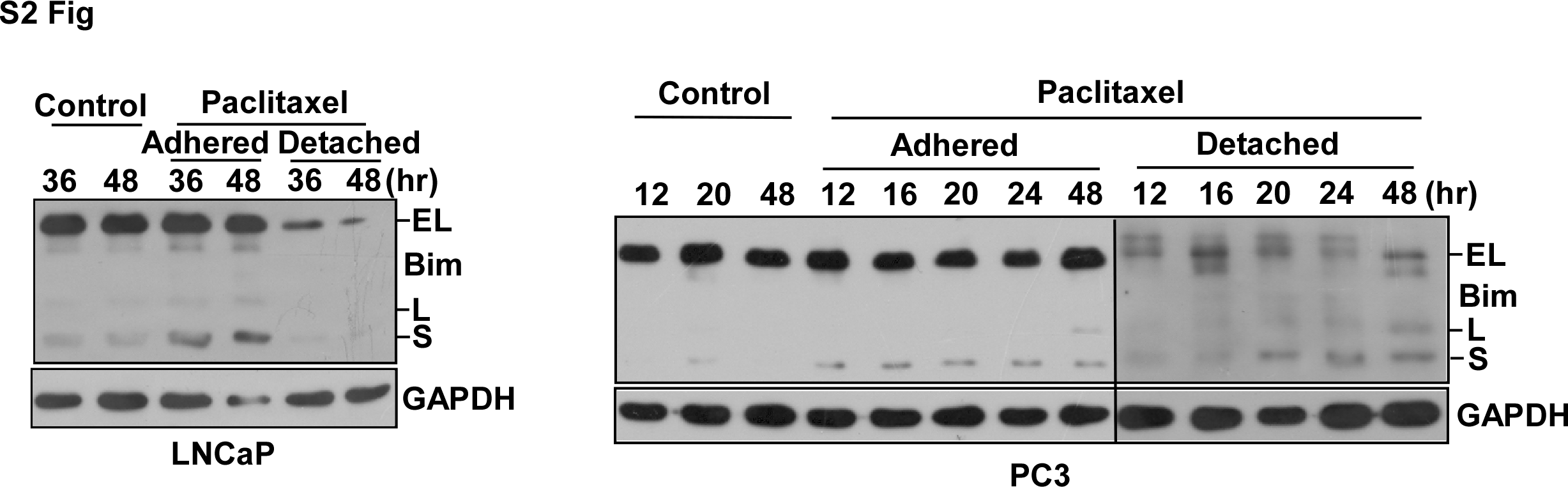

Supplement: S2 Fig — Immunoblot analysis of cell lysates from the adhered or detached fraction of LNCaP cells or PC3 cells after paclitaxel treatment through the time courses as indicated, for the detection of Bim. (TIF) [file pone.0120913.s002.tif]

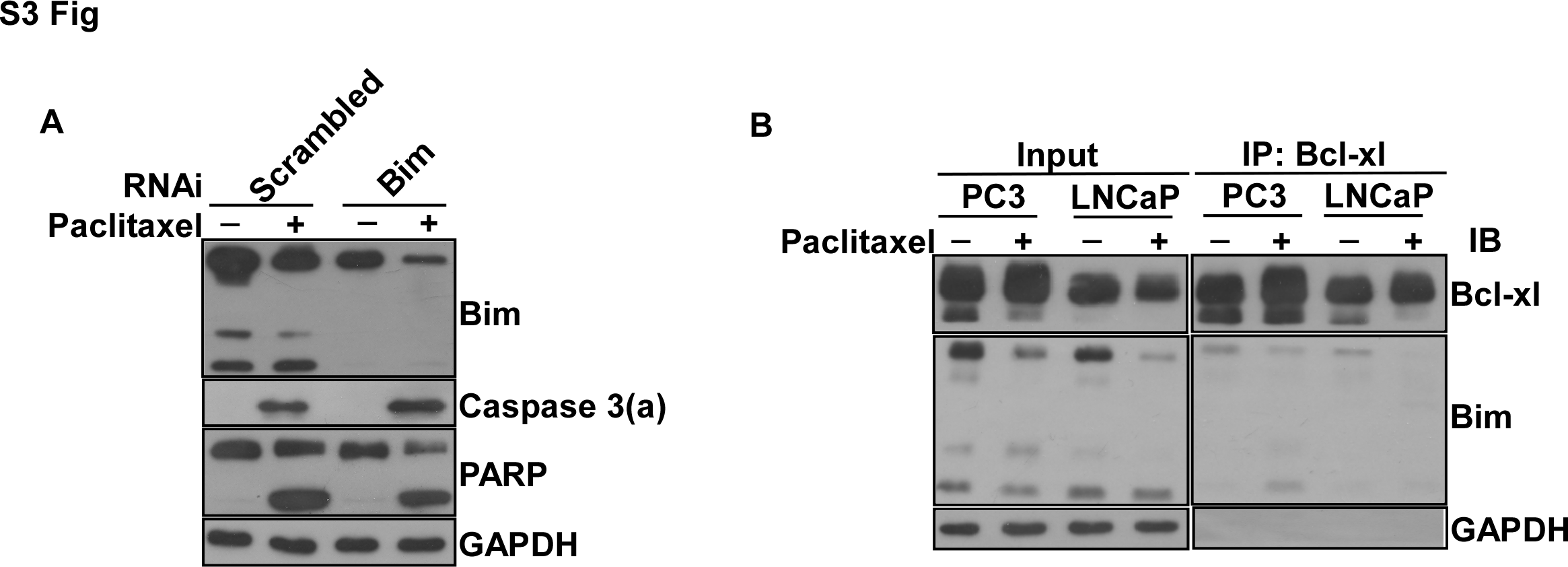

Supplement: S3 Fig — A) Immunoblot analysis of cell lysates from Bim knockdown of LNCaP cells after paclitaxel treatment. B) Immunoblot analysis of immunoprecipitates of Bcl-xl antibody for the cell lysates of LNCaP cells or PC3 cells treated by paclitaxel. IP: immunoprecipitation. IB: immunoblotting. (TIF) [file pone.0120913.s003.tif]
